# Retraction Notice

**DOI:** 10.4269/ajtmh.22-0524ret

**Published:** 2023-07-05

Peng H, Wang H, Guo X, Lv W, Liu L, Wang H, Cheng P, Liu H, Gong M, 2023. In Vitro and In Vivo Validation of *CYP6A14* and *CYP6N6* Participation in Deltamethrin Metabolic Resistance in *Aedes albopictus. AM J Trop Med Hyg 108(3):* 609–618. doi: 10.4269/ajtmh.22-0524. PMID: 36746656; PMCID: 9978559.

The above article, published in the *American Journal of Tropical Medicine and Hygiene*, has been retracted by the Journal.

After publication, an expert in the field identified numerous problems with this manuscript, including questions regarding appropriate methodology, inaccuracy of reported results, and, specifically, implausibility regarding the reported identification of deltamethrin metabolites. A second expert concurred with these impressions. Therefore, with serious questions about the methodology used, results presented, and conclusions made, the manuscript has been retracted after publication.

